# Supplementary material for: High performance magnesium-based plastic semiconductors for flexible thermoelectrics
Source: Nat Commun. 2024 Jun 14;15:5108. doi: 10.1038/s41467-024-49440-5 (PMC11178910; doi:10.1038/s41467-024-49440-5)
Supplement: Supplementary file 1 — Supplementary Information [file 41467_2024_49440_MOESM1_ESM.pdf]

Supplementary Information

High performance magnesium-based plastic semiconductors for flexible thermoelectrics

Airan Li[1], Yuechu Wang[1], Yuzheng Li[1,2], Xinlei Yang[1], Pengfei Nan[3], Kai Liu[1], Binghui Ge[3], Chenguang Fu[1,*], Tiejun Zhu[1,2,*]

[1]State Key Laboratory of Silicon and Advanced Semiconductor Materials, School of Materials Science and Engineering, Zhejiang University, 310058 Hangzhou, China. [2]Shanxi-Zheda Institute of Advanced Materials and Chemical Engineering, Taiyuan 030000, China. [3]Information Materials and Intelligent Sensing Laboratory of Anhui Province, Key Laboratory of Structure and Functional Regulation of Hybrid Materials of Ministry of Education, Institutes of Physical Science and Information Technology, Anhui University, Hefei 230601, China. [*]Email: zhutj@zju.edu.cn; chenguang_fu@zju.edu.cn. These authors contributed equally: Airan Li, Yuechu Wang

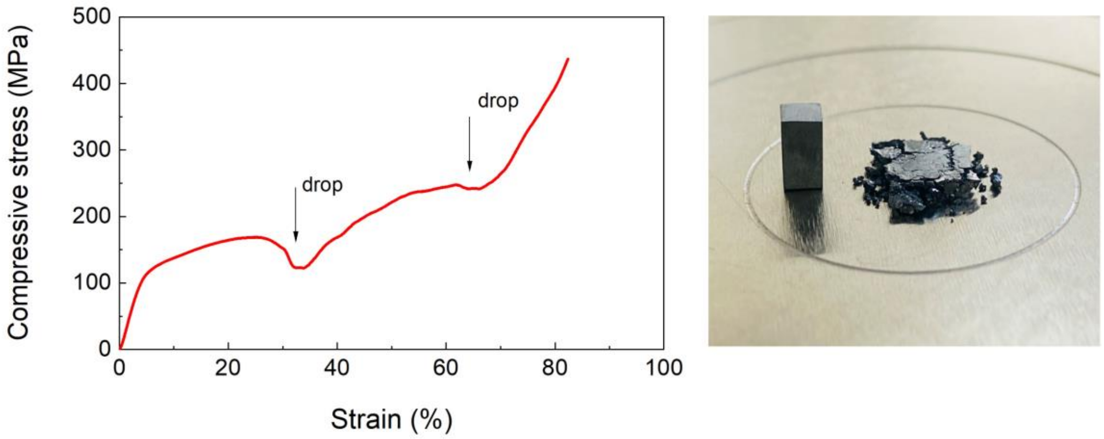

Supplementary Fig. 1 Compressive strain-stress curve of polycrystalline Mg_3Bi_2 with its optical image.

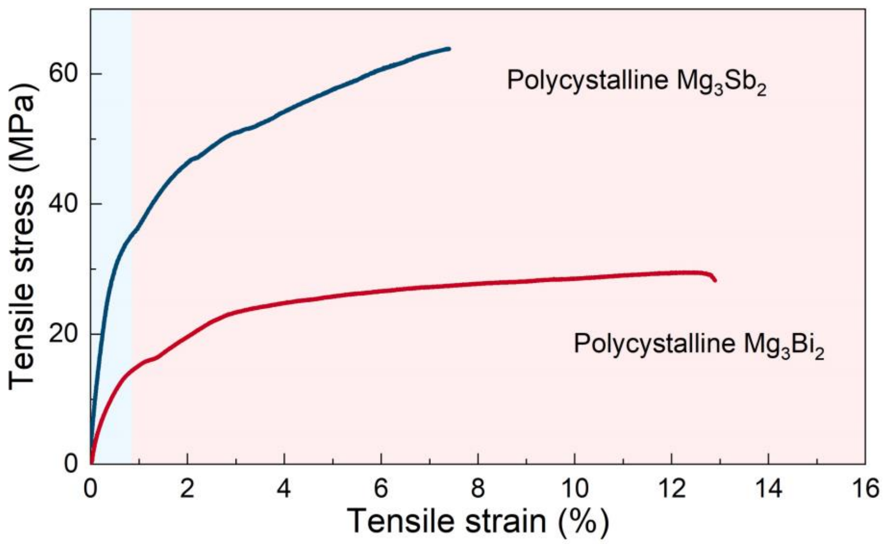

Supplementary Fig. 2 Tensile strain-stress curves of polycrystalline Mg_3Sb_2 and Mg_3Bi_2.

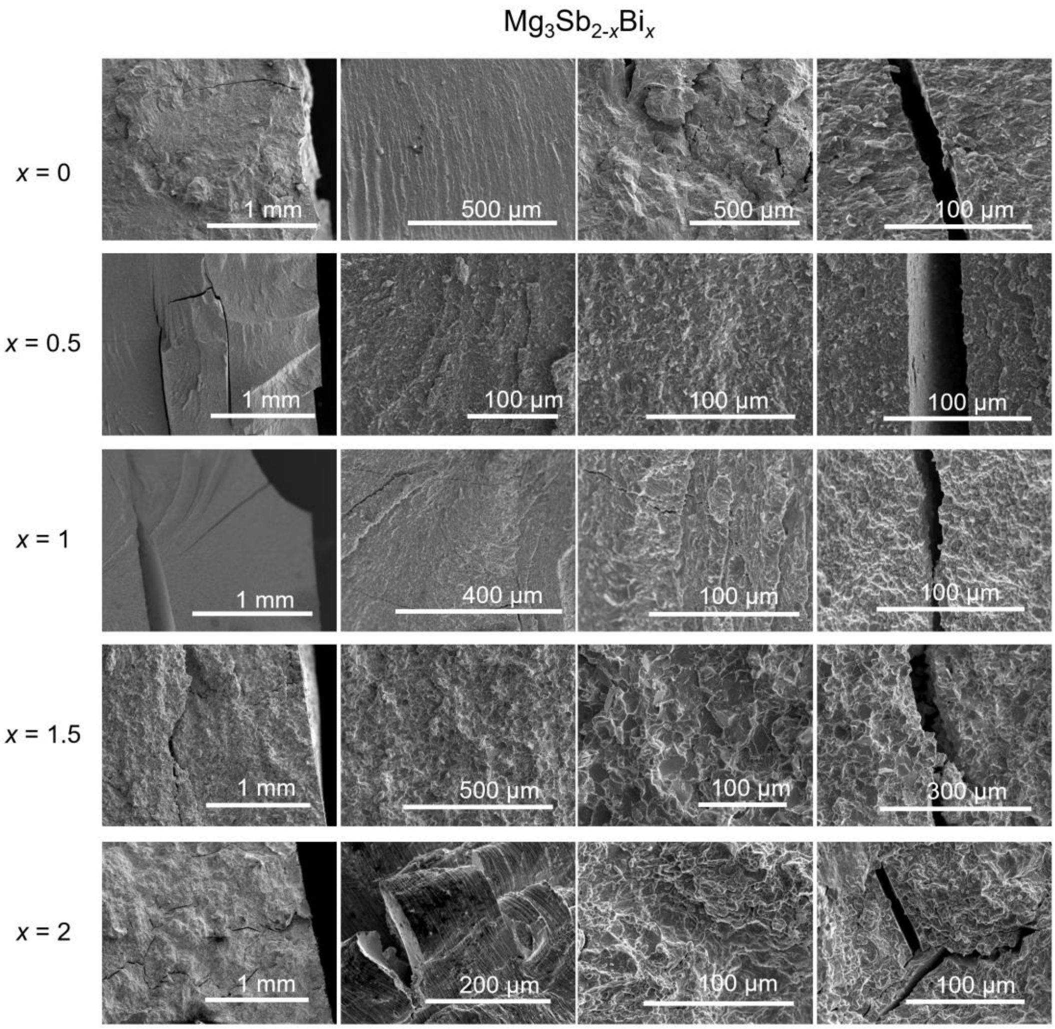

Supplementary Fig. 3 Fracture surface morphology of polycrystalline $Mg_3Sb_{2-x}Bi_x$ after compression.

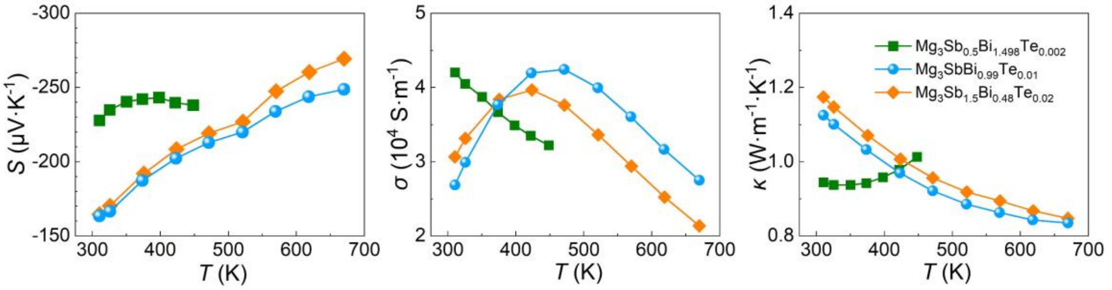

Supplementary Fig. 4 Temperature dependence of Seebeck coefficient *S*, electrical conductivity σ and thermal conductivity κ of polycrystalline Te-doped $Mg_3Sb_{2-x}Bi_x$: $Mg_3Sb_{0.5}Bi_{1.498}Te_{0.002}$, $Mg_3SbBi_{0.99}Te_{0.01}$, $Mg_3Sb_{1.5}Bi_{0.48}Te_{0.02}$.

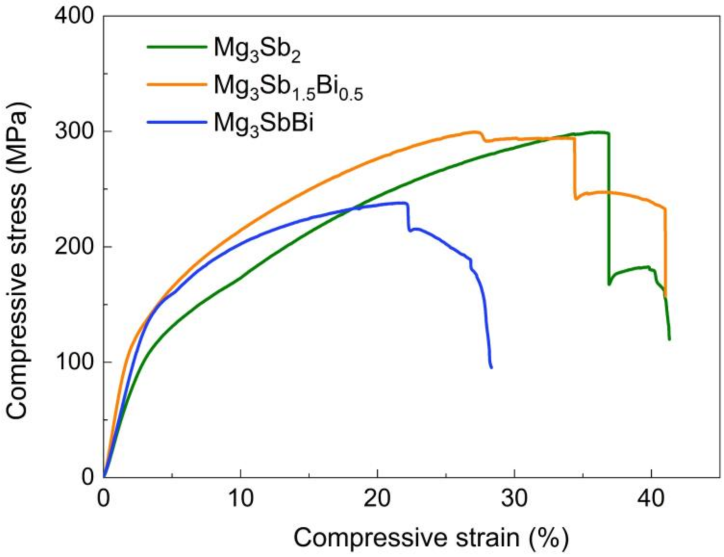

Supplementary Fig. 5 Compressive strain-stress curve of p-type polycrystalline Mg_3Sb_2, $Mg_3Sb_{1.5}Bi_{0.5}$, Mg_3SbBi without doping

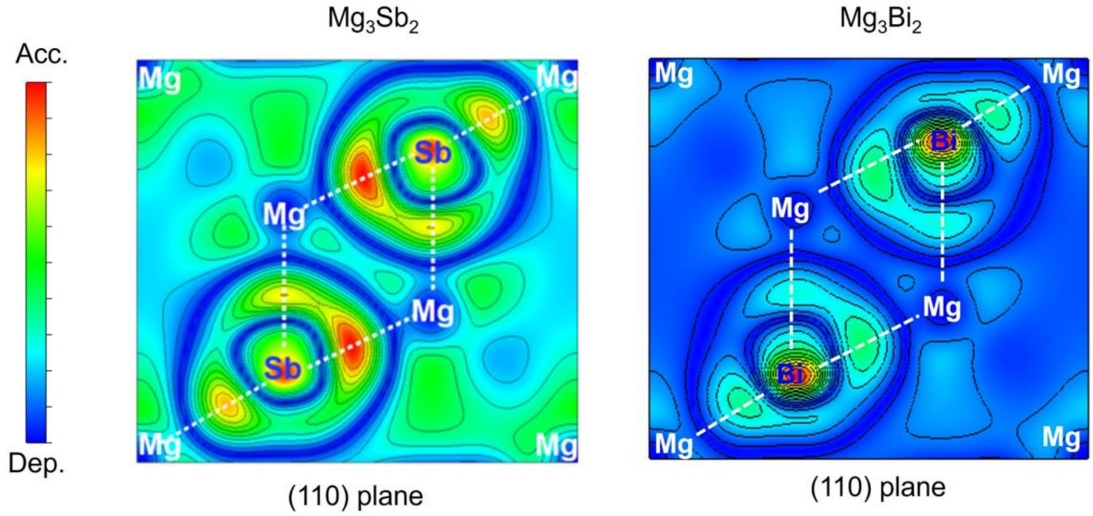

Supplementary Fig. 6 Charge density differences of Mg_3Sb_2 and Mg_3Bi_2. The localized electron accumulation can be found between the Mg and Sb/Bi atoms.

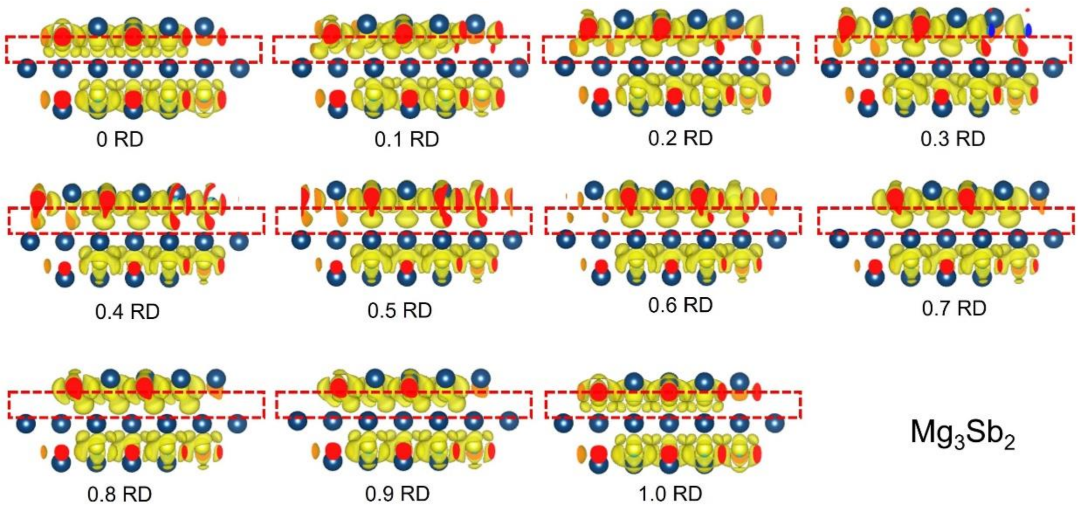

Supplementary Fig. 7 CDDs of Mg_3Sb_2 at different relative displacements (RD) along the [100](001) slip direction.

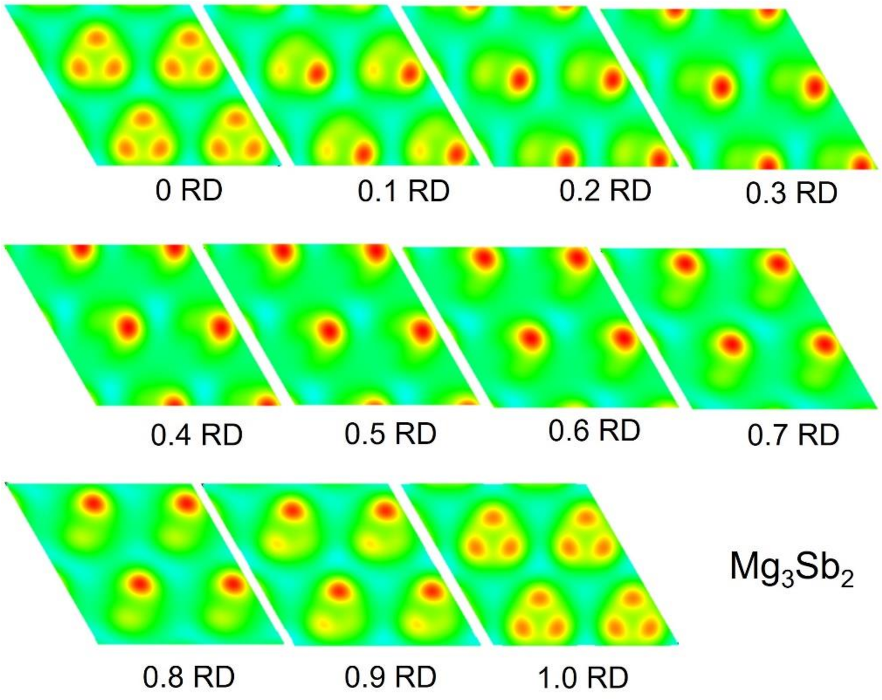

Supplementary Fig. 8 CDDs projection on (001) plane of Mg_3Sb_2 at different RD along the [100](001) slip direction.

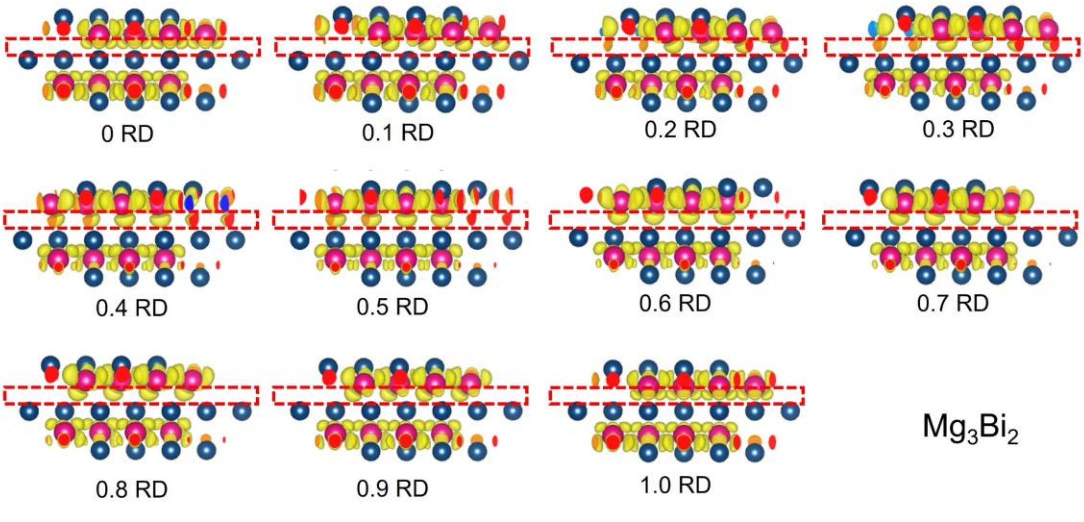

Supplementary Fig. 9 CDDs of Mg_3Bi_2 at different relative displacements (RD) along the [100](001) slip direction.

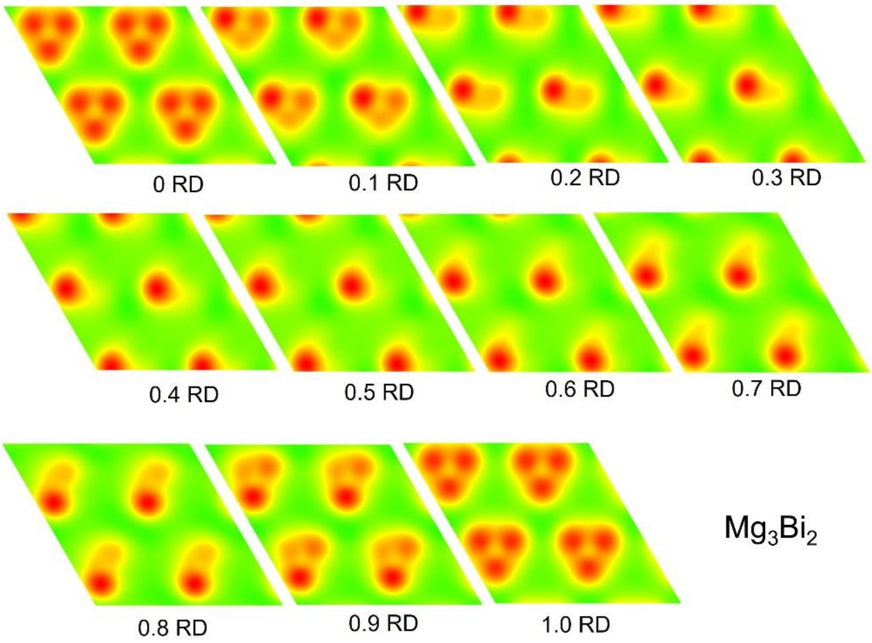

Supplementary Fig. 10 CDDs projection on (001) plane of Mg_3Bi_2 at different RD along the [100](001) slip direction.

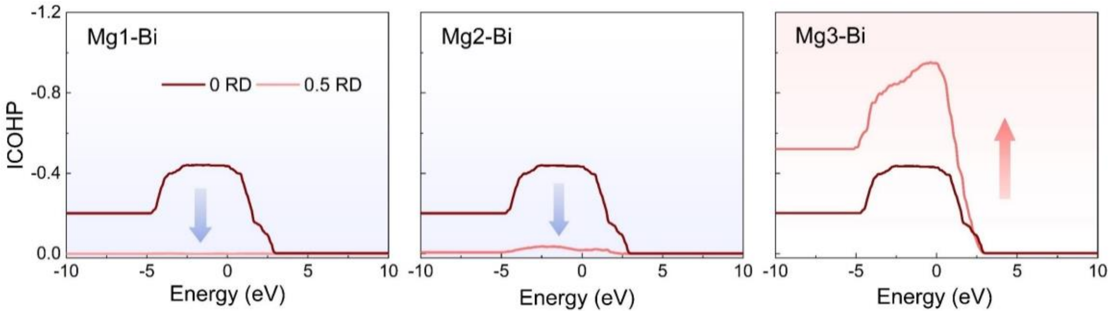

Supplementary Fig. 11 ICOHPs of Mg1-Bi, Mg2-Bi and Mg3-Bi with RD = 0 and RD = 0.5.

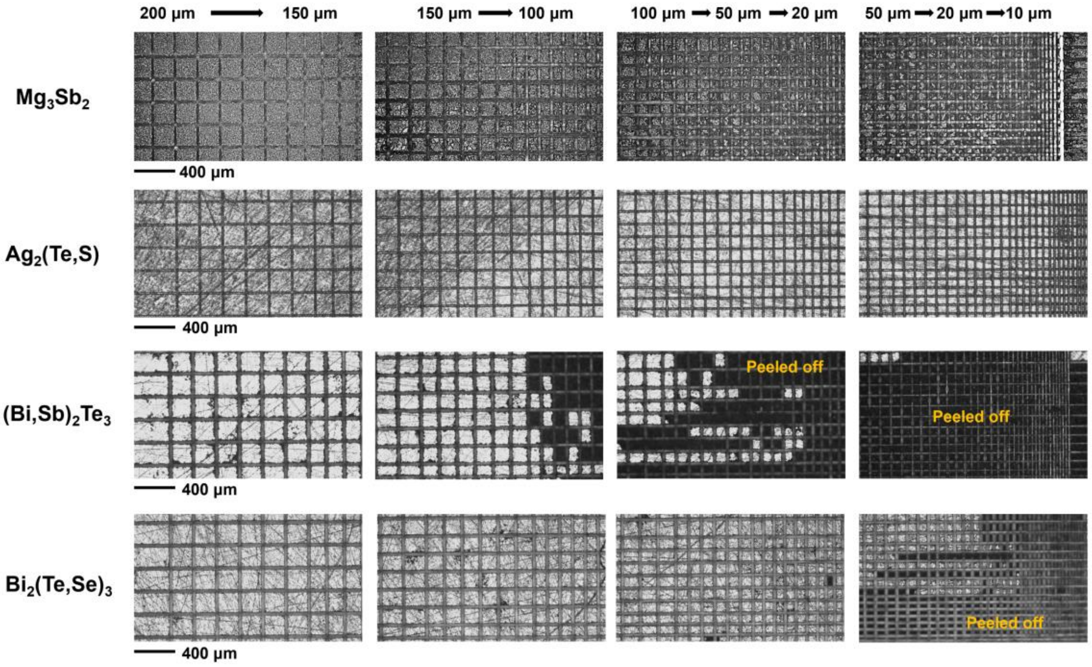

Supplementary Fig. 12 Dicing images of polycrystalline Mg_3Sb_2, Ag_2(Te,S) and $(Bi,Sb)_2Te_3$, and $Bi_2(Te,Se)_3$ with different dimensions.

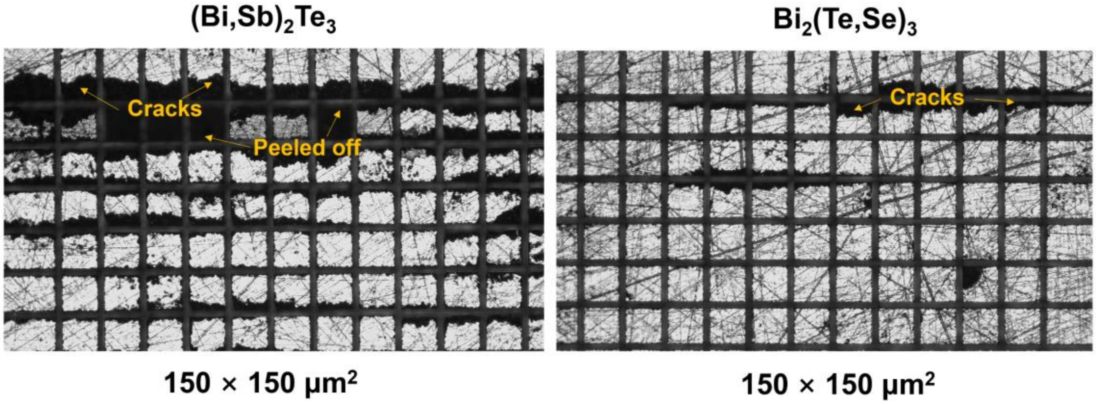

Supplementary Fig. 13 Dicing images of $(Bi,Sb)_2Te_3$ and $Bi_2(Te,Se)_3$ with dimension of $150 \times 150\ \mu m^2$.

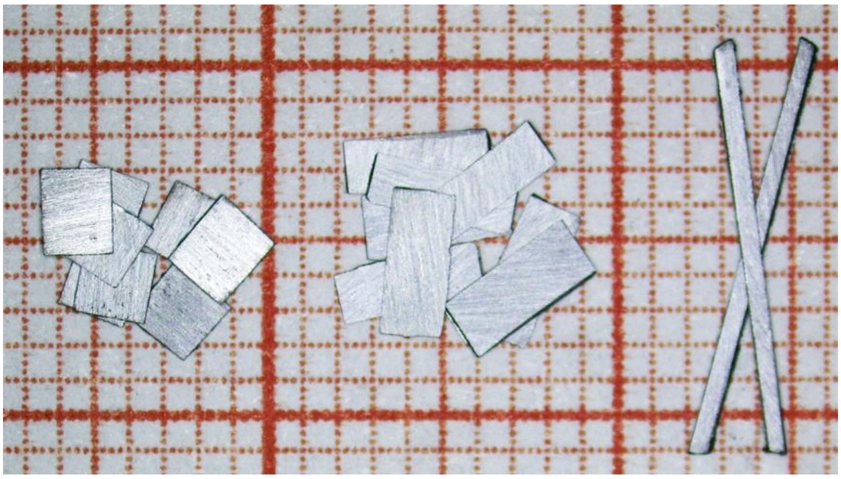

Supplementary Fig. 14 Different dimensions of polycrystalline $Mg_3Sb_{0.5}Bi_{1.498}Te_{0.002}$ by cutting.

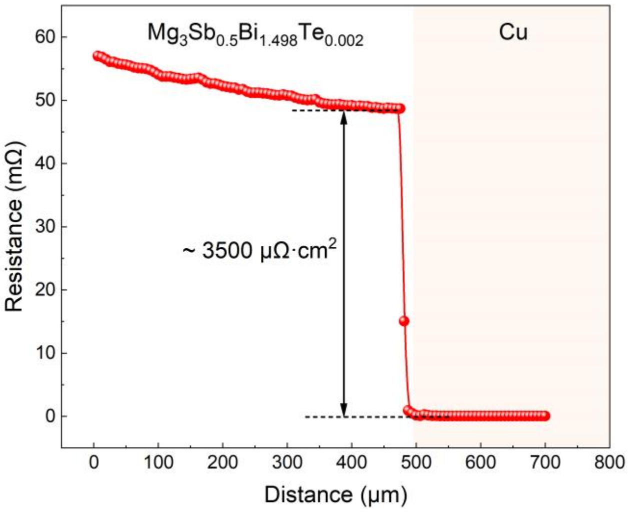

Supplementary Fig. 15 Contact resistance between $Mg_3Sb_{0.5}Bi_{1.498}Te_{0.002}$ and Cu electrode.
